# Characterization of Biological Resistance and Successful Drug Resistance Control in Medicine

**DOI:** 10.3390/pathogens8020073

**Published:** 2019-05-31

**Authors:** Rudolf Fullybright

**Affiliations:** Department of Applied Research, Applied-Research Center for True Development, Montréal, QC H1W 0A3, Canada; fullybright@gmail.com

**Keywords:** pathogen, antimicrobial, antibiotic, drug, resistance, infectious, design, pharmaceutical, combination, threshold, law

## Abstract

It has now been a century that drug resistance has been getting worse in human infectious diseases medicine. A similar trend is observed in veterinary medicine and agriculture. The successful control of drug resistance requires an understanding of biological resistance in general, as a phenomenon taking place in nature. Once we have understood the main characteristics of biological resistance and how it operates in nature, we can then apply that new understanding to its subset that drug resistance in human medicine is. Possession of such an edge can also lead to the successful control of resistance in veterinary medicine, in agriculture, and in other settings of resistance activity by biological organisms. Based on biological resistance data from human medicine, veterinary medicine, and agriculture, some of the fundamental characteristics of resistance as a natural process displayed by all living organisms are established. The consistent, common features characterizing the data are exploited, as is a mathematical model depicting how biological resistance strengthens in living organisms. It is found that biological resistance in general, and drug resistance in particular, is a phenomenon governed by at least two laws: the First Law of Resistance, requiring a threshold to be met before resistance can be prevented and the Second Law of Resistance, causing resistance to strengthen to infinite levels if unstopped. Inference is thereafter made as to the drug design strategy required for the successful control of resistance in medicine. To that end, the blueprint currently applied in the design of infectious diseases drugs needs revising.

## 1. Introduction

Drug resistance—or antimicrobial resistance—is the phenomenon by which pathogens destroy the effectiveness of drugs. The end result is always increased morbidity or death of the patient. Deaths from drug resistance are on the rise worldwide. Resistance-related deaths worldwide in the coming decades are expected to reach several million *per year*, *every year*. Today, resistance to antibiotics has become the word’s number 1 medical emergency. While every approach to overcome this threat has so far failed, this paper introduces a new approach and new insights that are expected to take us to the solution.

Encountered both in human and veterinary medicines, drug resistance has become a hallmark of infectious diseases—with important clinical and public health consequences. Beyond medicine, however, resistance is also encountered in agriculture [1]. In both medicine and agriculture, the trend has been worsening over the past several decades, has reached crisis proportions, and is showing no sign of abating [2,3,4]. In spite of the multiple strategies devised and implemented by the medical community so far, the situation has only been leading to increasing losses. So much so that, in agriculture, resistance has now been recognized as a serious threat to both animal welfare and crop production [5,6]. In medicine, and in Europe, more than 25,000 people die each year as a result of antibiotic resistance [7]. In the United States, 20,000 people die each year because of the methicillin-resistant *Staphylococcus aureus* (MRSA) bacteria alone [8]. In fact, overall antibiotic resistance in the US is already killing more people than car accidents, prostate cancer, and AIDS (the Acquired Immune Deficiency Syndrome) combined [2]. And the trend continues to rise. The attendant economic losses are no less staggering. As an example, the 6-year economic burden associated with *S. aureus* infections in the United States is more than 14 billion USD (US dollars)—a loss of more than 2 billion USD *every year* [9]. And this is exclusive of deaths and economic losses due to any other pathogen in any other country. Worse yet, similar trends are observed in other countries around the world.

It is safe to expect that our ability to overcome drug resistance and attain a successful control of pathogen organisms depends to some extent on our understanding of the intrinsic characteristics of biological resistance as a process inherent to nature. Once that understanding has been acquired, we will be a step closer to devising a strategy which will allow a successful control of resistance in human medicine, in veterinary medicine, in agriculture, and beyond.

For better resistance quantification and prediction purposes, we have previously broken drug resistance down into subsets of resistance termed “*resistance layers”* (cf. Supplementary Materials Table S1 of Reference [10]). Then, we have presented statistical models to predict the rate of new resistance emergence and cumulative resistance build-up across the quasi-totality of infectious diseases medicine—as represented by an aggregate of 118 pathogens [11]. The models make it possible to estimate our level of preparedness face-to-face with drug resistance from pathogens. Model-based predictions reveal that we are not nearly in a position to successfully contain resistance, neither now nor in the future [12]. Here, we show that biological resistance in general, and drug resistance in particular, is a natural phenomenon governed by at least two laws. We then infer the drug design requirement necessary for the successful control and abatement of resistance in human and veterinary medicines.

## 2. Materials and Methods

The First Law of Resistance is derived from the characteristics of resistance as a process that takes place across the entire biological realm and meets the definition of *law*. This is supported by the fact that every living organism, when threatened, puts out resistance in an attempt to self-preserve. That makes resistance a phenomenon not confined to medicine alone but, instead, everywhere present in nature. The First Law of Resistance holds an intrinsic corollary derived as a consequence of the First Law itself. Considering the Corollary to the First Law, the drug design requirement for a successful control of resistance in medicine logically ensues. The Second Law of Resistance is derived from a simple but accurate mathematical model (a function) which gives an account of the intensity reached by resistance interactions occurring between a biological organism and an opposing living or non-living entity, the manifestation of resistance necessarily requiring two participants. The value taken by the mathematical limit of that function for unlimited, never-ending interactions defines the Second Law of Resistance in the biological realm.

## 3. Results and Discussion

### 3.1. Characterization of Resistance: The First Law of Resistance

#### 3.1.1. Overview

Because drug resistance strengthens exponentially and faster than we can produce drugs [11], human life loss and economic losses from both medicine and agriculture can only be expected to also rise exponentially. For example, data have shown that MRSA-related deaths in the US have increased by more than 50% over just a 6-year timespan, while the estimated number of MRSA-related hospitalizations has more than doubled over the same period [13]. Although the exponential nature of the losses may not yet be apparent from this limited data pertaining to MRSA in the US alone, this strong rise in figures should be expected to eventually display an exponential trend. Chances are the exponential nature will be seen more clearly as more data become available over the years. In fact, confirming the worsening trend, drug resistance is now slated to take a minimum of 10 million human lives every year by 2050 [3]—roughly the same as the World War II (WWII) death rate. The bad part is that, because the losses are continuously rising, the number of lives lost will be even greater than WWII death rate in the years beyond 2050. And although some observers have contested the yearly death toll of 10 million lives by 2050, they still admit that there are both a large clinical and public health burdens associated with AMR (antimicrobial resistance), that this burden is likely to increase over time, and that urgent action is required [14]. More than the yearly 10 million deaths figure, therefore, what matters the most is the characteristic feature that antimicrobial resistance and the burden associated with it are increasing and will continue to increase into the distant future. Consequently, even if the yearly toll of 10 million deaths is not yet reached at the present time and will not be reached by 2050, it will still be reached at some point in time in the future as the situation continues to worsen.

Economically, on the other hand, the losses have also been showing a strong rise. For example, estimates of the extra cost due to MRSA infection as opposed to a methicillin-sensitive *S. aureus* infection range from 3000 USD to 35,000 USD [15,16,17], suggesting that MRSA infections cost patients and hospitals an extra 830 million USD to 9.7 billion USD in 2005 alone—not including indirect costs related to patient pain, illness and time spent in the hospital [13]. Furthermore, in agriculture, losses have also been on the rise: currently, herbicide-resistant weed alone is causing more than 17% loss in soybean yields and more than a 200-million-USD loss in both soybean and cotton in parts of the United States alone [18]. Observing the trend, it can be seen that the losses tend to parallel the strengthening of resistance in the organisms. Being a threat of that magnitude, it would be helpful to establish what some of the underpinning causes are.

Based on collected first-layer (i.e., monotherapy) resistance data from 1921 through 2007, covering roughly a 90-year timespan, 90 infectious diseases, 118 pathogens, and involving more than 300 molecules [11], the salient particularity can, in fact, be observed that a low, limited number of molecules is used in drugs targeting pathogens. Up to this day, no more than four molecules (of distinct chemical structures) have been used in combination drugs e.g., [19] and all such drugs have run into resistance from pathogens. But while a number of antimicrobial molecules used in modern medicine (all of which have run into resistance) are extracted from medicinal plants [20,21], medicinal plants have encountered *no* resistance from pathogens—no such report existing in the literature. As an example, the quinine molecule, extracted from *Cinchona* spp., has encountered resistance [22] and the artemisinin molecule, extracted from *Artemisia annua*, has also encountered resistance [23]; but neither plants has. In fact, an extensive database (e.g., PubMed) search of a pathogen having developed resistance to a medicinal plant yields no result. So, it can be stated that the root cause of the drug resistance problem lies at the extraction nexus—meaning that if we can decipher that nexus right, we will be in a position to derive hints which will be central to our efforts at subduing resistance.

#### 3.1.2. Features of the Process and First Law of Resistance

The United States Department of Agriculture’s James Duke phytochemical database [24], possibly among others, informs that medicinal plant species almost always contain a minimum of 50 to 100 molecules (of distinct chemical structures). These molecules are used simultaneously (i.e., together) against pathogens in holistic medicine—not one by one and not in any number less than the dozens which the herb naturally contains. The simultaneity of use in holistic medicine is facilitated by the modality of herbal tea: as the herb seeps, it releases all of its phytochemical content into the tea water which is then taken by the patient. Thus, by taking the tea water, the patient benefits from the synergistic action of not just 4 molecules but of all 50 to 100 molecules present.

Plants in general, and medicinal plants in particular, have been around for millions of years [25] and so have pathogens [26]. Throughout this time, medicinal plants have been interacting with and fighting off a variety of diseases and pathogens, both in their natural environment and in human patients. This continued, millennia-long interaction ought to have favored the rise of resistance in the pathogens against the plants. Yet, resistance to a medicinal herb has never been known or reported anywhere. In fact, all medicinal herbs from prehistoric times, formerly effective against specific pathogens, continue to be effective against the same target pathogens today. For example: *Panax ginseng*, used throughout the last 5000 years for a variety of pathogen-related conditions, continues to be effective today [27,28,29,30]; *Artemisia annua*, which has been used to treat malaria for more than 2000 years in Asia [31], continues to be effective today [32,33,34,35]; the North American *Sambucus racemosa* continues to be effective against the respiratory syncytial virus [36]; while the North American *Ipomopsis aggregata* continues to be effective against the influenza virus in spite of its long history of use [36]. Again, the only reports available are those rather informing that medicinal plants of times past are still effective against their respective target pathogens today. However, pathogen resistance has consistently negated every plant-extracted or lab-synthesized antimicrobial used in combination drugs of no more than four molecules. This trend has stayed consistent in pathogens over the past 100 years—since the first report of antibiotic resistance occurrence in 1921 [37]. This consistency has been to the extent that it is now expected that pathogen resistance to antimicrobials “will arise naturally” [38,39,40,41]. Comparison of the scheme used in holistic medicine versus the one used in modern medicine reveals, however, that the one single and salient element of difference resides in the number of molecules combined: the number of molecules combined being extremely low in current modern drugs.

Similar phenomena occur in other realms of life. In particular, beyond human medicine, drug resistance occurs and is observed in veterinary medicine as well. Along that line, veterinary medicine has been dealing with its fair and strengthening share of resistance. For example, there are reports of resistance against benzimidazoles, levamisole, pyrantel, and macrocyclic lactones in *Trichostrongylides* (nematodes) in small ruminants (sheep and goats) as well as in *Strongylides* in horses, while resistance against ivermectin is now rising in cattle [5]. However, beyond being just a question of infection of farm animals, nematoid resistance to pharmaceuticals has become a serious problem in many parts of the world, a major problem in veterinary medicine, and a serious threat to both animal welfare and agricultural production [6]. Furthermore, infection in these animals is not “infection” as we traditionally understand it to mean—as being caused by unicellular (single-celled) pathogens in medicine. Instead, these infections are resistant infections on a higher scale because the involved resisting biological agents are more complex organisms (than unicellular pathogens are). In addition to resistance by more complex organisms, resistance by unicellular pathogens which would have been treated with a limited number of isolated molecules, as in human medicine, is also common in veterinary medicine e.g., [42]. Such pathogens belong to the bacteria kingdom, while nematodes belong to the animalia kingdom. However, resistance by organisms of the fungi kingdom [43] and of the protist kingdom [44] is also known to occur. Overall, therefore, resistance of biological organisms to limited control means (in this case, molecules) is evident in human medicine, in veterinary medicine, and in multiple kingdoms; the totality of those organisms spanning the fungi, bacteria, protozoa, and other kingdoms.

Far from being confined to those kingdoms, nevertheless, resistance is manifest in the plant kingdom as well. This is evident in agriculture. Contrary to resistance in medicine (human and veterinary), resistance in the plant kingdom, as reflected by agriculture, although it remains, as in medicine, a devastating phenomenon with strengthening losses, is interesting in that it is multi-faceted. In fact, resistance in agriculture takes at least four forms: (1) resistance of plant organisms to artificial chemicals of human origin, (2) resistance of plant organisms to natural chemicals of animal origin, (3) resistance of animal organisms to artificial chemicals of human origin, and (4) resistance of animal organisms to natural chemicals of plant origin. We note that, for simplicity, “animal origin,” as used here, includes the bacteria, chromista, fungi, and protozoa kingdoms.

More specifically, and to illustrate with examples, resistance of the tomato plant to phyto-pathogens is known to occur [45]: a case of plant resistance to natural chemicals of animal origin; resistance of plants to molecular isolate forms of herbicides is known to occur [46]: a case of plant resistance to artificial chemicals of human origin; resistance of insects to molecular isolate forms of insecticides is known to occur, this type having been occurring since 1908, from the San Jose fruit pest *Quadraspidiotus perniciosus* to lime sulfur as a chemical agent [47,48]—a case of resistance from an animal organism to artificial chemicals of human origin—and, finally, resistance of the *Helicoverpa zea* bollworm to the cotton plant is also known to occur [49]: a case of resistance from an animal organism to natural chemicals of plant origin. Furthermore, resistance within the animal kingdom proper, as illustrated by resistance of animal pests such as rats to molecular isolate forms of pesticides, also occurs in agriculture and is a familiar challenge.

None of those forms of resistance, however, is any less common than the others, all forms being rampant [3,50]. Consequently, resistance in agriculture is one that is *not* confined to just one kingdom. Not only does it manifest within each respective kingdom, it also crosses border from one kingdom to another and vice-versa—straddling any two kingdoms at a time. Additionally, it is worth noting that, beyond those four types, a fifth type of resistance occurs in agriculture, even in the complete absence of a chemical attack. That is the case of a plant developing resistance to the *mechanical* action of a pest feeding on its leaves [51]. This one type of resistance is important—because it shows that the development of resistance by a biological organism is not necessarily mediated by chemicals as other types of resistance, as in medicine, would have us believe. What this means is that resistance as a process can be elicited through means other than chemicals or molecules. Resistance, factually, therefore, is simply a defensive mechanism which, meant to protect the biological organism, can take any form and is not restricted to chemical processes alone. As we look across the various realms of biological life with its multiple kingdoms, we see that living organisms, no matter their size or biological complexity, have the capacity to deploy resistance to any oncoming life-threatening agent, the success or failure of which resistance in preserving the biological organism’s life is function of the strength of the oncoming life-threatening agent. Resistance, therefore, is everywhere prevalent in nature, occurs in a variety of ways, and turns out to be a natural process arising spontaneously in biological organisms whenever a situation of threat arises.

Considering the chemical richness of medicinal plants and the fact that no such plant is known to have encountered resistance from pathogens up to this day, in spite of their having controlled and interacted with a diversity of pathogens for millions of years, and further considering the fact that not more than four molecules (of distinct chemical structures) have been used in combination drugs, all of which have run into resistance from pathogens, it transpires that the hypothesis or proposition can be advanced that the low number of control means (in this case, molecules) used in human and veterinary medicines to control pathogens is the facilitator of drug resistance emergence and that the identical process used in the control of weeds in agriculture is equally the facilitator of weed resistance emergence. However, this resistance-favoring condition of insufficient control means, encountered in human and veterinary medicines as well as in weed control in agriculture, is also a probable justifier of resistance development in other settings, as in the case of an animal organism’s resistance to natural chemicals of plant origin or a plant organism’s resistance to natural chemicals of animal origin.

It is therefore observable that, in the case of man-induced resistance in agriculture and in medicine, the organism targeted (the plant (e.g., grass) or the pest (e.g., rats)) is sought to be controlled by a low number of molecules. Consequently, the approach used in human medicine, veterinary medicine, and agriculture is one and the same, and the limitation on the number of control means remains the single salient characteristic common to those three areas of endeavor which are all grappling with resistance. Resistance, therefore, occurs beyond human medicine, crops up in veterinary medicine, is rampant in agriculture, crosses boundaries between realms of living organisms from one kingdom to another, vice versa, thereby encompassing all biological life forms as found throughout the kingdoms and has been occurring consistently, albeit unintendedly, ever since the first control experiment of a biological organism in agriculture, in 1908—more than 100 years ago [47,48].

By definition, a law is “a statement of fact, deduced from observation, to the effect that a particular natural or scientific phenomenon always occurs if certain conditions are present” [52]. On the other hand, a law equally is “a statement that describes invariable relationships among phenomena under a specified set of conditions” [53], as it also is a statement of an order or relation of phenomena that, so far as is known, is invariable under the given conditions [54]. Here, we are in the presence of a natural phenomenon (resistance development as a result of controlling a biological organism with limited control means) which, albeit unintendedly, has been repeatedly tested for more than 100 years of experimentations, the outcome of which (rise of resistance) has remained invariable and the same throughout all experimentations—consistently delivering resistance. Experimentations total thousands of repetitions from human medicine to veterinary medicine to agriculture (we have reported 731 pathogen-control experiments in human medicine alone [11], while more data are available out there in human medicine and further more are available in veterinary medicine and agriculture), with a consistent outcome validated by more than 1100 cases of first-layer (monotherapy) resistance and more than 15,000 cases of second-layer (dual therapy) resistance from human medicine alone, while more cases exist in human medicine and further more exist in veterinary medicine and agriculture; with no human interference of any kind, involving thousands of teams of researchers from around the world (we have reported 731 teams of researchers for human medicine alone [11], while more teams of researchers exist in human medicine and further more exist in veterinary medicine and agriculture); all teams reporting the same and consistent result of resistance development against a limited, low number of molecules used in human medicine, veterinary medicine, and agriculture—and so for more than a hundred years. Note, therefore, needs to be taken that hundreds of teams reporting the same and consistent result of resistance development for more than one hundred years effectively precludes any possibility of human interference with the experimental design.

Knowing that the chemical richness of medicinal herbs far exceeds the low number of molecules used in human medicine, in veterinary medicine, and in agriculture and that, as such, medicinal herbs, continuing to be effective up to this day, have encountered no resistance from pathogens throughout their millions of years of interaction with pathogens and through their millennia of continued containment of a variety of pathogen-induced diseases in humans; and knowing that all experimentations have been conducted with the *same*, *unique*, and *salient* experimental design characterized by the use of a low number of control means with the goal of containing a biological organism, from unicellular pathogens to lower and higher animals, then on to lower and higher plants; and being the case across boundaries of biological species within any given kingdom and further being the case across kingdom boundaries between any two of the six kingdoms, from the bacteria to the protists to the chromista to the fungi to the plantae and to the animalia kingdoms [55], thus encompassing the entire manifest realm and full spectrum of biological organisms, there occurs *consistent invariability*, which, in turn, allows *definite predictability*—as reflected by the more than 100 years of consistency. However, the consistent invariability and definite predictability features of the proposition make it a *law*, because that is the definition of *law* [52,53,54]. The consistent invariability and definite predictability features of the proposition making it a *law*, we present the law of resistance that follows.

##### The First Law of Resistance

“In the control of natural biological organisms, resistance will arise if control means are less than a given threshold.”

**Figure 1:**
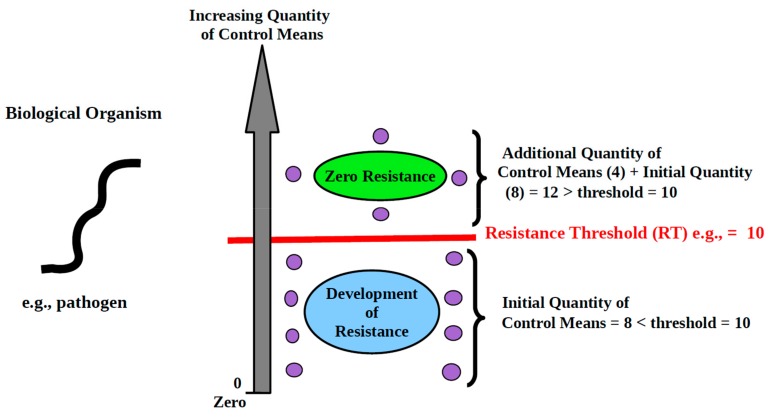
Schematic depiction of the First Law of Resistance as applied to infectious diseases. Legend: As the quantity of control means used remains less than the threshold, resistance arises in the biological organism. However, resistance vanishes once additional control means are brought in and cause the threshold to be met. Each purple circle represents 1 control means (in this case, a molecule).

The Law results from the fact that biological organisms are equipped with the natural instinct of self-preservation and will use any loophole in the control system to evade attack and survive. Consequently, control means must *always* be *at least equal* to a given minimum which, in effect, constitutes a threshold. Operating below the threshold inevitably allows resistance to arise—cf. schematic depiction of the First Law of Resistance, Figure 1. In that context, with control means at sub-threshold level, even escaping (fleeing) becomes a resistance mechanism: a way of making the control ineffective.

Illustration: We need to control mouse infestation. There are 10 mice to be caught in an area. The control means we choose to use is mouse traps. By definition, each trap can only catch 1 mouse. So, the threshold of the control means, which we will call “resistance threshold,” is 10. If we make use of 9 traps or less, we will run into resistance. Ten or more traps will allow catching all the mice and prevent resistance from arising. If we can only get 9 traps, or if we have more than 9 traps but choose to use only 9, then 1 mouse will escape control, will settle elsewhere, and reproduce. Then, we are back to square one. In attempting to control biological organisms, the threshold *must* be met. To avoid resistance, control means must *always* be *at least equal* to the required threshold.

The First Law of Resistance governs resistance manifestation in all areas of biological activity including infectious diseases medicine and agriculture.

#### 3.1.3. Corollary to the First Law: Resistance Thresholds

The First Law informs that the use of partial, insufficient control means in an attempt to restrain a biological organism will inevitably lead to resistance. A corollary to that Law is that for any scenario in which biological organisms are sought to be contained, the control means must be of a given minimum quantity if resistance is to be prevented. We define Threshold of Resistance in the control of biological organisms:

Resistance Threshold (RT): the least quantity of control means necessary to prevent the development of resistance.

The requirement of a resistance threshold applies to all cases and scenarios of resistance manifestation in the biological realm, in medicine and beyond. In infectious diseases medicine, in particular, the means used to control pathogens is molecules. Therefore, the resistance threshold in infectious diseases medicine is the least number of molecules to be combined in a drug in order to prevent pathogen resistance from arising.

### 3.2. Successful Control of Resistance: Drug Design and the First Law of Resistance

The observation that medicinal plants have shown, up to this day, no sign of falling victim to resistance in spite of their having been used in the battle against all kinds of pathogens since prehistoric times [56,57] is significant. Knowing that all medicinal plants of times past continue to be effective against their target pathogens today and recalling that a great deal of manufactured drugs is derived from medicinal plants [20,21,58,59], while all such drugs have run and continue to run into resistance from pathogens, the question arises as to what the flaw is that allows pathogen resistance to arise with so much certainty against manufactured drugs. Consideration of the Corollary above and the First Law of Resistance suggests that resistance arises because the required resistance threshold is not being met.

One of the characteristic features of drugs currently designed to combat pathogens and a few other non-infectious diseases such as leukemia [19] is that they contain a limited, low number of molecules compared to the internal chemicals-rich environment of medicinal plants. In fact, only one molecule has been used in most manufactured drugs until the recent past. For synergistic reasons, however, combinations of no more than four molecules, identified as “highly active therapy” [60], are now being tried. The fact remains, nevertheless, that all known combination drugs of up to four molecules which have stayed in use long enough have run into resistance from pathogens e.g., [19,61]. But, although the debate is still on and we therefore currently do not have an exact model describing synergistic processes in biological organisms [62], we still know, while allowing for limited antagonism, that, based on the empirical observation that the effect of the sum is greater than the sum of the effects, synergism increases as the number of combined molecules increases. Consequently, as combinations of up to four molecules have consistently run into resistance, we ought to be asking ourselves what the probable number of molecules to be combined in order to overcome resistance is. 

The US Department of Agriculture’s phytochemical database [24] gives an exhaustive (or nearly exhaustive) list of the phytochemicals present in a wide array of medicinal plants. And although the functionality of each of those phytochemicals is not currently known, it is still observable that medicinal plants contain a minimum number of chemicals almost always upwards of 50. Knowing that synergistic effects cause combination drugs with a greater number of molecules to be more potent and, therefore, more able to resist resistance than drugs with a lower number of molecules, and knowing that, in spite of their having interacted with a variety of disease-causing pathogens for millions of years, medicinal plants have encountered no resistance from pathogens, it can be inferred that, although some limited antagonism could be occurring in there, the assemblage of roughly 50 different molecules or more in medicinal plants delivers enough synergistic effect to allow medicinal plants to shun resistance and prevent it from arising.

Knowing that the combination of up to four molecules in combination drugs still yields resistance and that about 50 molecules shun resistance, it becomes evident that between 4 and 50 is located the minimum number of molecules to be combined in drugs in order to prevent resistance from arising. This number is the resistance threshold (RT) to be met in the drug design process by the pharmaceutical industry for purposes of pathogen resistance prevention. This means that if we combine molecules in a number less than this RT number, we can expect resistance to arise, while a combination of a number of molecules at least equal to this value will allow resistance to be prevented. This RT number is the limit number of molecules to be combined in drugs and determines whether we will run into resistance from pathogens or not.

Here, note needs to be taken that the hypothesis according to which “molecules are inducers of resistance in pathogens” was tested and was found to be true [11]. However, at this point we can see that this hypothesis was true only because the molecules were combined below threshold. In other words, the hypothesis is not true broadly. “Broadly,” here, meaning “for any number of molecules, even beyond threshold.” In fact, if the hypothesis were true broadly, then medicinal plants, with their often more than 100 molecules, would have encountered resistance from pathogens a long time ago. However, medicinal plants have encountered no (zero) resistance in their millennia of existence and interaction with pathogens. Therefore, the hypothesis is *not* true broadly. That is to say, the hypothesis is true *if and only if* the number of molecules combined is less than the threshold. Consequently, we are encountering resistance because the drugs we currently design are not meeting the threshold.

Beyond, and in addition to, the fact that there is no report in the literature of existence of resistance to a medicinal plant, we are, in the accompanying Supplementary Materials file together with the references supporting it [63,64,65], giving mathematical proof by contradiction that medicinal plants have encountered no resistance from pathogens, along with a list of more than 100 select medicinal plants (Supplementary Table S1 therein) with the respective target pathogens the plants are still effective against, the published literature references indicating that the said plants are still effective against those pathogens, and the years the effectiveness findings were reported. It can be seen from the information presented in that Supplementary Materials that medicinal plants have encountered no resistance from pathogens and are still effective against their target pathogens today.

The foregoing means that we are not encountering resistance because of a supposed abuse or misuse of antibiotics as is currently thought [66]. In fact, populations worldwide have been accused of favoring the rise of resistance through the misuse and abuse of anti-infection drugs, physicians have been accused of favoring the rise of resistance through the supposed over-prescription of drugs, and the pharmaceutical industry has also been accused of not developing enough drugs to circumvent existing resistance. But we now know that none of those accusations is true [12].

Another area of misunderstanding in the establishment of the causes of resistance emergence is that of counterfeit drugs. For a long time, counterfeit drugs have been signaled as being one of the causes of resistance development in pathogens [67]. However, pursuing this avenue for purposes of resistance control cannot deliver any actionable results in terms of effectively subduing resistance because counterfeit drugs are *not* the root cause of resistance emergence in pathogens. Not that this is in defense of the counterfeiting of drugs, but the objective analysis of the resistance situation with the goal of identifying and removing errors in our understanding of it requires the recognition that counterfeit drugs are not the foundational culprit in pathogen resistance emergence. In fact, only after we have acquired a truly clear view, an untainted picture of the situation we are facing can we hope to be close to determining the path that will liberate us and effectively move us forward. In that context, admission now needs to be made that, contrary to what has been held so far, counterfeit drugs are not nearly at the helm of resistance generation by pathogens. True, sub-concentrations of active principles in a medication are a facilitator of resistance emergence, and it is also true that damages wrecked by counterfeit drugs can be numerous (potential death due to the disease progressing, reduced confidence in the healthcare system, economic losses for the pharmaceutical industry, and so forth [68]). However, as far as resistance is concerned, the problem we are facing is more than just that of sub-concentrations of a few molecules identified as “active principles.” As we aim at tackling resistance broadly, there is a need to reckon with the fact that even drugs that are not counterfeit and are genuinely manufactured by the pharmaceutical industry have also encountered (and continue to encounter) resistance [69,70]. And, as seen, the molecule-dependency factor alone is causing that resistance to rise exponentially [12], logically meaning that the current fight against counterfeit drugs, even if successfully conducted, will contribute little, if anything, to controlling resistance because resistance stemming from drugs genuinely manufactured by the pharmaceutical industry is rising and is rising exponentially. Therefore, although the marshaling of resources for the fight against counterfeit drugs may be useful for other purposes, the expectation that it will help combat or contain drug resistance is not warranted. Instead, we are encountering resistance because of a conceptual flaw in the theoretical foundation supporting our design of anti-infection drugs and, thereafter, in the drugs designed on the basis of that theory. Once this flaw is rectified by meeting the threshold and other relevant concepts (e.g., synergism/antagonism, as covered below), resistance will vanish, whether there is abuse or not, misuse or not.

Nonetheless, increasing the number of molecules to be combined in order to meet the threshold cannot be done haphazardly. Hints are needed. Along that line, the new understanding needs to be acquired that synergism does not have to involve only molecules beneficial to the health of the patient. Molecules that are toxic or otherwise antagonistic on their own can quickly become beneficial to health once they enter combinations involving other molecules. One example is the glycyrrhizin molecule (from *Glycyrrhiza uralensis*, commonly known as “licorice”) which is known to be toxic on its own [71]. On the other hand, *G. uralensis* contains at least 179 separate chemicals [24]. However, *G. uralensis* has been in use since prehistoric times in the form of herbal tea [28], a modality which allows the additional 178 molecules to be simultaneously ingested by the patient, through the tea water, together with glycyrrhizin. Yet, no case of toxicity resulting from *G. uralensis,* from glycyrrhizin, or from any other naturally-occurring molecule therein, is known to this day when the herb is used in that fashion [28]. Therefore, glycyrrhizin is toxic alone; true, but when combined with the other molecules, it is no longer toxic. This is an example of a molecule which is detrimental on its own but then, once combined with other molecules, becomes beneficial—or harmless.

Generally, we have to recall that the concept of toxicity arises typically only as a given threshold of a compound is crossed and that the dose makes the poison [72]. The notion of categorizing some molecules as fundamentally beneficial or useful and, therefore, allowable into drugs and others as fundamentally deleterious or useless and, therefore, to be excluded from drugs needs rectification. Such categorization is erroneous and prevents us from benefiting from certain possibilities. In fact, there are examples of foods with naturally-occurring toxins but whose consumption is still safe. For example, prussic acid glycosides are toxic compounds occurring at low levels in the fruits of many fruit trees, but the consumption of which fruits remains harmless [72]. So, the inclusion of toxic or otherwise deleterious molecules in combination drugs should not be seen as anathema—as is currently the case. Depending on the role they play, their presence can be necessary.

The point is that synergism is not the fact that all molecules involved in a combination drug ought to each be beneficial, as is currently understood in medicine [73,74]. This important point was proven and reported by a number of investigators [75], possibly among others. Instead, synergism is rather the fact that some of the molecules involved in the combination might have deleterious or antagonistic effects on their own but then, once introduced into the combination, contribute to limiting, if not preventing, other negative effects (beyond the immediate goal of curing the disease) or to boosting the effect of some of the other molecules they are blended with, or both.

Practically, to illustrate, that means the following: molecule A kills pathogen *p* but delivers tiredness as a side-effect, molecule B also kills pathogen *p* and delivers no side-effect, molecule C delivers an antagonistic effect to killing the pathogen but prevents or reduces tiredness. Our goal is to kill pathogen *p* which is making the patient sick. Therefore, the application of our traditional understanding of synergism would have us suggest that molecules A and B ought to be combined, which is acceptable. However, synergism is also about including molecule C into the combination. Why?—Because although it is an antagonistic molecule which works counter to the immediate goal of killing the pathogen, it also delivers another effect which is the suppression of the undesired tiredness coming from molecule A, and, further, its antagonistic effect in working counter to the goal of killing the pathogen can potentially be contained by the dual synergistic output from molecules A and B. Synergistically, that also means that we can expect the healing effect from the combination of dual molecules A and B to be greater than the counter-effect provided by the single C molecule. The foregoing means that molecule C can also be included in the combination involving molecules A and B. However, we have been excluding antagonistic molecules from our drug engineering processes so far.

Our traditional practice, up to this point, has been to design drugs by including only literally beneficial molecules in synergistic combinations [75], but we now need to grow past that understanding and come to grips with the synergistic relevance of molecules other than literally beneficial ones. This means that beyond the fundamental goal of curing the target disease, control of undesired effects should also be aimed at, as well as potentiation of the beneficial effects. The inclusion of molecules to attain this additional purpose of control of undesired effects and potentiation of beneficial ones can only lead to an increase in the number of molecules bound to enter the combination. As the number of molecules entering the combination increases, we will get closer and closer to the required threshold. As we get closer and closer to that threshold and eventually cross it, resistance can be expected to vanish. On the other hand, it can further be expected that increasing the number of molecules entering the combination will allow quorum quenching—the inhibition of quorum sensing, even in resistant pathogens—to take place [76].

What all of that means is that once we begin seeing synergism no longer as molecules working together to suppress just the target disease but rather as various molecules coming together to suppress both the disease and undesired side-effects (including resistance, which can be seen as a side-effect resulting from the use of the drug) as well as potentiating the beneficial molecules involved in the combination, we are a step closer to successfully controlling resistance. This understanding is key because if we can design drugs that are molecules-rich enough (rich enough to meet the threshold), then we can be sure that they will deliver such a strong synergistic output that resistance initiation by pathogens cannot even start, to begin with. This conceptual understanding will be essential to our efforts at controlling resistance. Although our current grasp of synergism is such that it can be likened to a potential madhouse [62], the realization that synergistic output aimed at curing the disease but also at the prevention of undesired side-effects and potentiation of beneficial molecules increases the number of molecules entering the combination, thereby favoring the meeting of the threshold, will be useful. Along that line, investigators have now shown how an example of potentiation in the case of tuberculosis has not only called for additional molecules in the combination drug but has also prevented resistance emergence in the TB cells [77]. That finding supports the concept presented here.

The overall picture which transpires from the foregoing is that we have been encountering resistance only because we have been operating below threshold, at a low level of synergistic output, as reflected by the low number of molecules we currently combine. In fact, we currently combine 04 molecules as a maximum, while medicinal plants combine, say, 40 at a minimum. So, it can be seen that we have been encountering resistance at least in part because we have not been making advanced use of synergism. Proceeding that way prevents us from meeting the threshold requirement, thereby allowing resistance to arise.

In practice, the RT number could vary from pathogen to pathogen, and such variations should be expected. However, a definitive threshold necessarily exists which, we can expect, is greater than all the individual pathogen thresholds. This definitive threshold needs to be investigated, possibly experimentally, if drug resistance is to be successfully contained. Doing so will probably call for a number of experimental challenges to be overcome. For example, in the course of engineering such a threshold-satisfying drug, individual molecules but also molecule-molecule interactions, safety, toxicity, clearance, side-effects, potentiation, mechanisms of action, and so forth will need to be investigated for both the combination and sub-combinations of the molecules involved. It is no exaggeration to expect this to be a major biochemical engineering challenge. However, this level of difficulty is not reason for not attempting this investigation, because solving an advanced challenge (this new approach to drug design) is likely to be more rewarding than solving an easier one (our current approach) if the solution to the former is potentially useful while the solution to the latter is potentially unavailing [78]. Nevertheless, regardless of the level of challenge it may provide, it can be expected that only after this investigation has been integrated into our drug design processes can we hope to begin seeing the fading away of the drug resistance phenomenon.

The necessity of such an investigation is reflected by the fact that resistance will continue to increase to infinite levels if we maintain current practices (cf. Section 3, Second Law of Resistance, below). On the other hand, resistance being a generator of stress, we can never handle an infinite quantity of it because, sooner or later, we will run out of resources (material and human). Also, note needs to be taken that these observations apply equally to agriculture. In fact, rising resistance in that department will end up making food production impossible, with the consequences we know.

The danger remains, however, that as we begin combining former resisted molecules into combination drugs, the potency of the resulting drugs will still be less than it could have been had each molecule been brand new. This is due to the fact that the pathogen would have already developed resistance to the individual molecules involved in such a combination. For example, assuming that the threshold stands at 10, if we consider 10 molecules the individual effectivenesses of which are already low due to the pathogen having already developed resistance to each of those 10 molecules, then, as we combine those molecules into a 10-molecule combination drug, the resulting effectiveness, although it can be expected to be greater than that of the individual molecules, will still be less than the effectiveness we would have derived had we combined only 10 non-resisted, brand new molecules. Therefore, although we may have understood that combination therapy practiced in a way to meet the threshold requirement is the way out of the drug resistance challenge, we still have only limited options if the molecules available in our armamentarium are already, each, negated by resistance—and this seems to be the case currently. Therefore, our best bet is to generate brand new molecules and to combine them into threshold-satisfying combination drugs so that pathogens would not have had the opportunity to initiate resistance to the individual molecules involved in those combinations. In that regard, novel approaches to discovering new molecules in an accelerated manner will prove useful [79,80]. Conversely, we will continue to undermine ourselves and to scupper our prospects if, as new molecules are discovered/conceived, we continue to combine them in ways that fail to meet the threshold.

In fact, the US National Institutes of Health and the European Commission, together, currently spend no less than 425 million USD every year on drug resistance control and tens of billions of dollars are now scheduled to be disbursed for the production of just 15 new antibiotics (which will trickle in, instead of coming as a pack) over the next decade [81]. However, without a redirection of efforts away from current drug design theory to the new understanding of combining newly-discovered molecules in ways that meet the threshold (so as to prevent resistance from arising in the first place), even such tremendous investments will eventually prove to have been to no avail, while resistance would have gotten even worse—molecules involved in sub-threshold combinations only causing resistance to get (exponentially) worse. The current sub-threshold approach to new infectious diseases drugs production is an important error which is costing lives. Why spend all that money on a yearly basis only to make the situation worse and worse each year? So much financial resources are disbursed yearly, only with the outcome of aggravating the situation more and more.—A catastrophic scenario. 

Cost-wise, the combination of a greater number of molecules in combination drugs will come to an overall production cost greater than the cost of producing current drugs. However, this does not literally translate into an unsustainably high cost, first because a combination drug so designed will probably find applications on more than one pathogen, as is currently the case—and this will contribute to keeping overall production costs down. Second, the long-term benefit of preventing the development of resistance *altogether* will generate important financial gains which will override any initial production costs, knowing that resistance is a generator of important (and worsening) financial losses [9,11]. As a result of those benefits, the scientific challenge associated with the investigation and production of threshold-satisfying combination drugs needs to be taken earnestly. On the other hand, although medicinal plants typically contain molecules in a number often greater than 50, such that the threshold is certainly located between 4 and 50, the said threshold might not be that high, considering that a species such as *Galium odoratum* contains just 14 molecules [24] and considering also that not all molecules found in plants have antimicrobial activity. Some molecules (as hormones) have other functions, such as growth regulation and those may have to be excluded from the total of often upwards of 50 found in medicinal plants, meaning that the threshold of antimicrobials that we need to attain in our henceforth novel combinations may, after all, not be very high.

Taken together, our theoretical framework on the basis of which drugs are currently designed requires revising and the precepts introduced here will help usher in a new era in drugs engineering.

### 3.3. Characterization of Resistance: The Second Law of Resistance

#### 3.3.1. Overview

By definition, resistance arises only as a result of an encounter between two opposing entities, at least one of which must be biological. In the interplay between those two opposing entities, the goal of each is to annihilate the other. In the particular scenario of drug resistance in medicine, a molecule and a pathogen are in opposition with each other, each seeking to render the other ineffective. However, beyond medicine, the same feature is observed in every resistance interaction occurring in the biological realm.

We define Entity 1 and Entity 2 as the two participants in the resistance interaction. Because the objective of Entity 1 is to suppress or weaken Entity 2 (and vice-versa), Entity 1 will continue to be functional and to bear down on Entity 2 as long as its final objective of suppressing or weakening the latter is not achieved. The end result is worsening resistance.

#### 3.3.2. Features of the Process and Second Law of Resistance

Biological science informs that any living organism has its life to preserve and is therefore equipped with a natural instinct of self-preservation. This allows the organism to self-defend in the face of danger. However, the natural instinct of self-preservation arises in the biological organism spontaneously and independently of the volition of the individual. The spontaneous and instinctive nature of the rise of self-preservation strategies, regardless of the will of the individual, is a key feature. As determined as Entity 1 is in annihilating Entity 2, the self-preservation strategies of Entity 2 also have an equal goal to reach: keeping the biological organism (Entity 2) safe and alive. As such, those self-preservation strategies will not relent as long as the opposing influence of Entity 1 remains active against Entity 2. In effect, we have a system in which two evolutionary forces are determined to oppose each other and to continue to oppose each other as long as the one opposed continues to exist. Being of an evolutionary nature, these forces are powerful. Because volition of the entities involved is irrelevant due to the instinctive nature of the self-preservation mechanisms and because each side is determined to attain its goal of destroying the other in order for it to continue to be, there arises within this interaction a very first feature which we can call “blind determination.” The second feature, which is of equal importance, is that the determination of each side to reach the goal it has set for itself leads to regular incremental rises in the overall amount of resistance prevailing in the system formed by the pair.

In this interplay between Entity 1 and Entity 2, therefore, *q* being the quantity of resistance initially put out by the initiator of the confrontation (say, Entity 1) and which is subsequently matched by an opposing resistance of equal intensity *q* deployed by the other participant (Entity 2), in the next stage of the ongoing interaction, Entity 1, determined, as it is, to reach its goal of destroying Entity 2, will have to increase its offensive output by a quantity *i* (*i* standing for “incremental” resistance). Or, else, we could witness the other scenario in which Entity 2 responds to the initial output *q* of Entity 1 with a counter-resistance level of *q + i* instead of just *q*, in which case Entity 1 will respond back by putting out an additional incremental resistance *i* (defensive mode: to suppress just the prior output *i* from Entity 2) or it could respond by putting out an incremental resistance amount of 2*i* (offensive mode: to suppress the prior output *i* from Entity 2 and then go on the offensive with an additional output *i*) which, in turn, will generate yet another incremental rise from the other participant, Entity 2. This interplay goes on until the system formed by the pair somehow breaks apart when the resistance level within it becomes excessive and unbearable for either participant.

The foregoing word-based description of the process by which resistance slowly creeps to high levels is easier visualized and modeled in mathematical form, mathematical models allowing a better tracking of processes. Translating, therefore, that process into mathematical form, we find that it is modeled with a linear, first-degree polynomial function:

*R*(*x*) = 2*q* + *xi* (1)

where:

*R*(*x*) is the quantity of resistance in the system at any given incremental rank, that is, *x* = 1st, 2nd, 3rd, …, or *n*th increment, in units of resistance [82];
*q* is a constant, the initial quantity of resistance (in units of resistance) deployed by the one participant who initiated the confrontation;
*x* is the number of incremental rises which has occurred since the beginning of the interaction. As a count, *x* is abstract (dimensionless);
*i* is an increment of resistance, in units of resistance [82]. As such, *i* is (and can only be) positive.

We observe that the mathematical expression above is one of two options. In fact, at each rank *k*, we know that the rise of resistance in pathogens is a process which is not instantaneous and rather takes time. For example, it has been found that a pathogen can take as many as 3 years (sometimes more) to display resistance to a new drug [83]. So, the quantity of resistance in the system at rank *k* which will take, say, three years to display its incremental rise in resistance, really depends on where one stands in measuring it:

(1) at the very beginning of those three years, the pathogen has not yet developed resistance. At that point, the quantity of resistance in the system is equal to the quantity of resistance which existed in that system at the end of the previous rank: rank *k* − 1.
(2) at the end of those three years, however, the pathogen has already generated its incremental rise in resistance for rank *k*. At that point, the quantity of resistance in the system is equal to the quantity of resistance in that system at the end of the current rank: rank *k*.

So, the mathematical model takes the form *R*(*x*) = 2*q* + (*x* − 1)*i* or the form *R*(*x*) = 2*q* + *xi* depending on whether one chooses to evaluate the amount of resistance in the system at the *start* or at the *end* of the incremental rise at each rank *k*, respectively. This observation for an incremental rise coming from the pathogen remains true and equally applies to an incremental rise coming from the pharmaceutical industry which also requires several years to develop and introduce a new drug to the market against the pathogens.

Here, the author has chosen to work with the model evaluating resistance at the *end* of each incremental rise. However, we note that regardless of the model one chooses to work with, the mathematical limit at infinity remains the same.

The above equation allows measuring how strong the resistance (*R*) has gotten in the interaction between the two parties. The strength of *R* depends on the initial amount of resistance (*q*) in the system, on how many incremental rises have occurred (*x*), and on the average value of each increment (*i*). 

Note: To simplify, *i* was taken in this model to be a constant which remains the same throughout the entire interaction between the two entities. However, for an accurate characterization of the process, note needs to be taken that *i* itself can vary in any fashion because it depends both on the individual and on the magnitude of the instantaneous response from each participant. Generally, *i* indicates the pace at which the process is evolving. This pace could be slow and *i* would be a small number; or the pace could be fast and *i* could increase as a power function, as an exponential function, or as any other rapidly-increasing mathematical function. Any rate is possible. The point remains that *i* can never be negative or equal to zero. The special case where *i* becomes zero marks the end of the resistance interaction.

However, should accurate quantification be sought, the *exact* value of *i* at each incremental rank, including from the very beginning of the interaction, needs to be considered. Then, we have:

(2) $$R\left(x\right)=\;\overset{x}{\underset{\;k\;=1\;}{\sum }}i_{k}$$

where: *k* designates a given incremental rank, *x* the highest rank of interest, and *i* as before.

In any case, as long as the system made up by the pair of opposing participants exists and is not yet split apart or destroyed by excessive resistance, *x*, the number of increments, will continue to increase. Then, the eventual quantity of resistance prevailing within the system made up by the pair is given by the limit of the *R* function as *x* tends to infinity. We have:

(3) $$\underset{+\infty }{\lim}R=\;\underset{x\to \;+\infty }{\lim}\left(2q+xi\right)=\underset{x\to \;+\infty }{\lim}\sum_{k=1}^{x=\;\infty }i_{k}=\;+\infty$$

This result means that once resistance begins, it keeps on increasing to infinite levels—if nothing stops it.

By definition, all living organisms are endowed with the natural instinct of self-preservation [84,85]. All living organisms being endowed with the natural instinct of self-preservation ensures, consequently, the occurrence of continued incremental rises in their resistance interactions. As a result, the two requirements for resistance to increase to infinite levels, if nothing stops it, are met for all biological organisms: (1) blind determination (to ensure their self-preservation) and (2) regular incremental rises (in the strength of the overall quantity of resistance in the system). The mathematical result above, therefore, remains true for all biological organisms involved in any resistance interaction—regardless of whether the said interaction is occurring between two opposing entities of the plant kingdom, between two opposing entities of the animal kingdom, or between any two entities of any two kingdoms (including the mineral kingdom, which is not a biological kingdom or kingdom of life). As such, the above result goes into effect every time resistance develops between any two opposing entities, affects all biological organisms, remains consistent across all types of resistance interactions, and is therefore *consistently invariable*. Being consistently invariable, the result is *definitely predictable*. However, as seen earlier, the consistent invariability and definite predictability features of the result make it a *law*, because that is the definition of *law* [52,53,54]. The consistent invariability and definite predictability features of the result making it a *law*, we present the law of resistance that follows:

##### The Second Law of Resistance

“Once begun, resistance increases to infinite levels if no process * occurs to stop it.”

* These processes include withdrawal or suppression (death) of either one of the participants involved.

Another such process, theoretically, is fitness cost: as the pathogen develops resistance to molecules, it is supposed to lose virulence or infectivity and should become less fit, that is, less capable of infecting new hosts. Therefore, it will be less likely to encounter a new molecule (as a cure taken by the patient) to which it will develop a new resistance. So, the tally of the total number of resisted molecules should slow down and should end up remaining static. However, we note that the contribution of fitness cost to the overall resistance reduction objective that we have is negligible and cannot be relied on. This is because resistance is known to have been growing worse while the total number of resisted molecules has also been rising. What that means is that if fitness cost as a damper of infectivity or virulence were important enough to have any noticeable impact on resistance in terms of lowering it, then pathogen resistance would have progressively slowed to the point of becoming remarkably small by now. However, pathogen resistance has not slowed at all and has, instead, been strengthening to the point of having now become a global crisis. Therefore, fitness cost as a dampener or slower of resistance is not an important parameter to rely on. This is due to compensatory mutations, which erase/override the loss incurred in fitness and compensate for it [86].

On the other hand, we note that the Second Law of Resistance is fully compatible with the proposed resistance measurement scale which now makes the measurement of infinite quantities of resistance possible [82].

Generally, Equation (3) means that the more incremental rises have occurred and the greater the average increment, the greater the prevailing amount of resistance in the system. Zeroing in on drug resistance, in particular, Equation (3) means that the more molecules (*x*) the pharmaceutical industry introduces to the market (and the greater the average incremental rise *i* which, in this case, is the resistance level, expressed in percentage, of the pathogen to the molecule), the more serious drug resistance will get and the tougher it will be to handle it. If this process does not stop because of some intervening factor, then it has the potential to increase to infinite levels, translating into an infinite number of resisted molecules. This reality is proven by the fact that in 1921, there was only 1 case of resistance, worldwide, all diseases confounded [11]. Then as we began manufacturing a new molecule to replace a previously-resisted molecule, we are now standing at a tally of more than 300 resisted molecules and counting—with no end in sight. Attempting to control resistance by continuously introducing a new molecule in order to bypass a pre-existing resistance is a wild goose chase with no end in sight. That means that drug resistance can only worsen and will reach infinite strengths if a way is not found to stop it altogether. The foregoing equally means that current resistance management strategies, which are designed to “contain” or “control” resistance, thereby allowing residual resistance to continue to exist, cannot meet the goal of stopping resistance altogether because such management strategies favor the continued supply of molecules to the pathogens by creating a loophole in the Second Law, loophole through which this residual resistance will later flare back up [12].

#### 3.3.3. Medical Implications of the Second Law of Resistance

A loophole is created in the Second Law through the existence of residual resistances. In fact, “Once begun” (in the statement of the Law) implies that existence of residual resistance is an indication that resistance “has (already) begun,” which is a violation of the non-starting condition of the Second Law. That is to say: In order *not* to reach infinite levels, resistance must *not* start, which means that the design of the drugs needs to be such that resistance *cannot* start. So, that resistance has started is a violation of the non-starting condition of the Law. This violation causes the Second Law to inevitably kick in—leading to continuously-increasing resistance. Unfortunately, up to this point, we have been satisfied with so called “low levels” of resistance (expressed in percentage) e.g., [87,88], viewing them as acceptable. This is a major mistake. We should not be content with those perceived “low levels” of resistance which, truly, are residual resistances. We should not be content because such low levels of resistance (which are residual resistances) are a harbinger of future trouble to come. Leaving residual resistances around hurts us precisely because they activate the Second Law and allow it to remain active—taking us to ever-increasing levels of resistance. In fact, as this “low level” resistance increases, however slowly that may be, we end up traveling the full reach of intra-molecular resistance (cf. Supplementary Materials Table S1 of Reference [10]). This causes us to end up in the range of 70s, 80s, and 90s % resistance. At that point, we will admit that the molecule has become totally ineffective and switch to a new molecule, thereby increasing cumulative or cross-molecular resistance by one unit (cf. Supplementary Materials Table S1 of Reference [10]). However, this new molecule will, as before, travel the full reach of intra-molecular resistance and will end up in the 70s, 80s, and 90s % resistance again, at which point we will recognize that it has become totally ineffective as before, will switch to yet another molecule, and the cycle starts over again. In the end, the cumulative number of molecules introduced to pathogens increases steadily and cumulative resistance also increases—but *exponentially* so. We cannot get out of this situation with that kind of procedure. Viewing low-level resistance as acceptable is a serious mistakebecause it favors that cycling process, which is taking us to infinite levels of resistance. By allowing residual resistance to exist, we have been playing the resistance game (by entertaining it). But the resistance game is a bankruptcy game and we cannot afford to play it. Either way, we will be conquered because of the infinite levels of resistance that this game is taking us to and which we cannot handle. A long-term strategy for the successful control of resistance (in medicine or agriculture) requires that we abandon that kind of approach, implying that the one-by-one/sub-threshold introduction of molecules to pathogens has got to stop. We have no choice—lest we sign our demise. And this characteristic feature equally applies to pest and weed resistance control in agriculture.

The fact remains that resistance, be it in medicine or agriculture, must be stopped totally. *Totally*. The least bit of it cannot be allowed to exist because, however small it may be at first, the Second Law is there and will amplify it. At this point, it can be seen that everything we are doing right now is wrong. Everything. These errors need rectification. The challenge is certainly high. Again, resistance must be suppressed totally because of the Second Law. And the way to suppress it totally is through rectification of the theoretical foundation supporting drug design, as seen.

**Figure 2:**
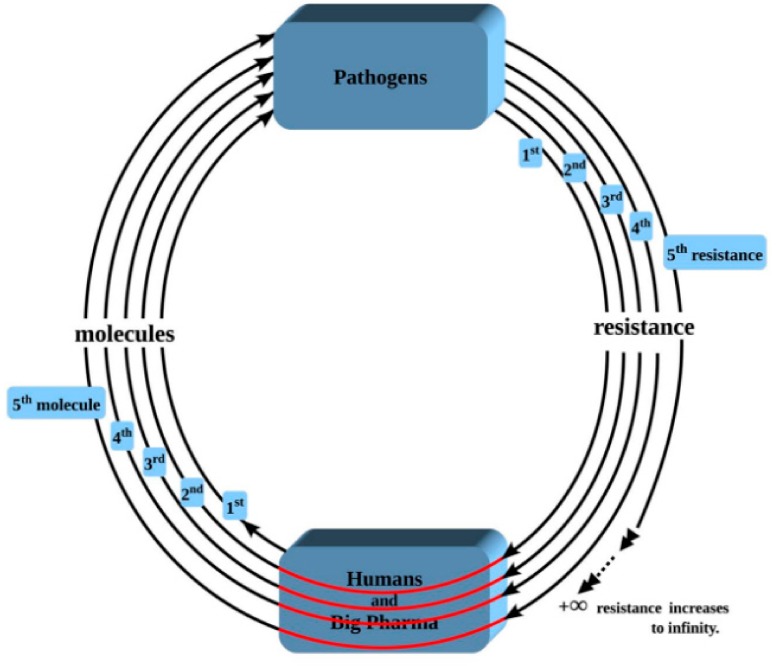
Depiction of the Second Law of Resistance as applied to infectious diseases. Legend: Through the patient, humans/Big Pharma supply the first molecule to the pathogens; then the pathogens return one resistance back to humans. This makes it 1 spiral with 1 resistance (innermost spiral). As resistance arises to that first molecule, the humans/Big Pharma complex is forced to introduce yet another molecule to the pathogens in order to circumvent/bypass/avoid that initial resistance; then the pathogens return an additional resistance back to humans. That makes it 2 spirals with 2 resistances. As of 2007, the count is standing at more than 300 resisted molecules [11]. As the spiral continues to be recycled over and over again, drug resistance, as represented by the total number of resisted molecules, increases to infinite levels, which we will not be able to withstand.

The Second Law of Resistance applies to all cases of resistance manifestation in the biological realm, including and beyond medicine and agriculture. However, as far as drug resistance in medicine is concerned, the Law shows that once resistance starts in a pathogen, the only outcome which can be expected is the strengthening of that resistance to infinite levels if nothing brings it to a stop. Based on this result, therefore, as long as we stay engaged on a path which gives rise to resistance development in pathogens, such as the path we are currently on, the hope to overcome resistance cannot and will not materialize, contrary to current expectations. Currently-held expectations are that if we follow certain safeguards, then we will overcome resistance. However, this result is saying that drug resistance, if not totally suppressed but allowed to exist the least bit-, cannot be contained and will only get worse, no matter what we do. Safeguards currently proposed for overcoming resistance include not using drugs longer than necessary, not using drugs when not necessary, not prescribing sub- or over-doses of drugs, reducing antibiotics use in farm animals (although this last suggestion has now been shown to have negligible impact [89]), and so forth. The problem is that each of those safeguards allows continued use of the said drugs, which implies continued interaction between drug and pathogen, even if at a reduced rate. By allowing continued use of the drugs and, therefore, continued interaction between drug and pathogen, each of those safeguards allows drug resistance to increase steadily, however slowly, to infinite levels, by virtue of the Second Law, in the sense that one resisted molecule will be replaced with a new molecule which will later become resisted, so forth. So, there is potential for steady increase in the total number of resisted molecules, thereby taking us to ever-increasing levels of resistance (cf. graphical depiction of the Second Law of Resistance, Figure 2).

The other complication which arises as part of this worsening of resistance is that, as more and more molecules become resisted as a result of our continued supply of newer and newer molecules to pathogens, there is going to also be a parallel and continued increase in the number of resisted but not-yet-created molecules due to entrained resistance [12], entrained resistance being favored by resisted molecules leading resistant pathogens to exhibiting cross-resistance to other molecules of the same class or to other molecules of totally different classes [90]. So, we will not only be dealing with known resisted molecules; we will also be dealing with resisted but not-yet-created molecules. This phenomenon narrows our options further.

The Second Law of Resistance explains the observed clinical fact that resistance cases have been getting tougher and tougher to treat e.g., Reference [91]. The rate at which this strengthening is occurring in infectious diseases medicine as a whole is given by the new resistance emergence and cumulative resistance build-up rate models earlier presented, which are exponentials [11]. So, drug resistance is not only strengthening, it is strengthening pretty fast. The course on which we have been, and which has been generating this strengthening of resistance, is therefore one which logically needs to be abandoned. If the current path is not abandoned and is rather maintained, then resistance can only worsen, no matter what we do and regardless of *any* safeguard we may conceive and implement. Practically, that means that if we continue to design drugs the way we are currently doing, then resistance can be expected to never vanish. The foregoing implies that a change of course is required so infinite resistance can be avoided. The new course of action, required for the successful control (i.e., suppression) of resistance, is given by the First Law of Resistance applied to drug design (Section 2, above).

#### 3.3.4. Select Illustrations of the Second Law of Resistance

A patient has tuberculosis (TB). The anti-TB drug is Entity 1. The biological organism, the TB bacillus, is Entity 2. The goal of Entity 1, which is under the volition of the patient or doctor, is to suppress the biological organism made up by Entity 2, the bacillus. As such, Entity 1 will continue to be present and functional in the patient’s system as long as Entity 2 (the bacteria) has not yet been fully destroyed. This is because the doctor will keep giving the drug to the patient until the latter is healed.

Although one may be tempted to think otherwise, volition is not involved here: the natural self-preservation instinct of the TB bacillus suppresses its volition and prevents that volition from manifesting. As well, the need for the patient to live or the requirement for the doctor to save his life (which, also, is self-preservation instinct) factually suppresses any volition or choice on the part of either the patient or the doctor. In spite of the fact that they are the ones who “choose” to send the drug into the patient’s system, this is a forced choice which is really not a choice. Volition is suppressed because both the patient and the doctor are determined or required to preserve human life. Therefore, the bottom line is observed that volition is excluded from the system and the system, made up by the two participants (the drug, controlled by the doctor or the patient and the bacteria), functions on the basis of the sole blind determination of the two opposing, evolutionary, self-preservation forces, opposing each other until the annihilation of one by the other. As a result, the first feature of the Second Law is satisfied.

However, the second feature, regular incremental rises, is also satisfied. Satisfaction of this feature is initiated by the bacteria which develops an incremental resistance *i* to the drug, while no resistance existed in the system before. At the beginning of the treatment, there was no resistance, so *R* = 0. However, at this point, when the bacteria develops its first incremental resistance, the amount of resistance in the system is equal to *i*: *R* = *i* (or *q*). Then, the doctor responds by increasing the dosage of the drug (which is a form of “resistance” to the resistant response from the bacteria) once s/he notices that the bacteria has developed resistance to the previous dosage. Consequently, there is another incremental rise *i* which, this time, comes from the doctor. At this point, we have a resistance intensity of *R* = 2*i* in the system, and the process proceeds similarly to reach *R* = 3*i*, then *R* = 4*i*, and so forth. This, in fact, is a recurrent theme in the treatment of infectious diseases: when physicians detect resistance while another drug is not yet available on the market, the first measure they find themselves taking is increase the dosage of the drug (to kill the pathogen). On the other hand, if a new drug were available, that drug would be more potent than the drug then in use. As a result, if the doctor chooses to switch to the new drug, s/he is still responding to the initial resistance output from the pathogen with an incremental rise *i* and the development of resistance by the pathogen to the new drug will create yet another incremental rise in the overall amount of resistance prevailing in the system, and the process continues. Therefore, either way, the second feature of regular incremental rises is satisfied.

Because the two features are satisfied and the process goes on repeating itself, we end up having to deal with greater and greater levels of resistance on the part of the bacteria (resistance strength [10]), which, on the ground, translates into our greater and greater difficulty in curing patients. Beyond tuberculosis, this factapplies to all infectious diseases.

Generally, the statement of the Second Law of Resistance can be seen in the resistance curves of the aggregate pathogens (new and/or cumulative resistance vs. time and/or molecules) as well as in the resistance curves of individual pathogens [11]. Beyond medicine, the Second Law can also be observed in the graph depicting the strengthening of weed resistance to herbicides in agriculture in the United States [92] or globally [93]. In each scenario, resistance is seen to be increasing indefinitely.

## 4. Conclusions

We have investigated some of the features characterizing biological resistance as a natural phenomenon displayed by all living organisms. It was observed that resistance manifestation in nature (in medicine and beyond) is governed by at least two laws and that resistance would not have arisen in the first place had the First Law not been violated. However, should the First Law be violated and resistance arise, this resistance has the potential of strengthening indefinitely—by virtue of the Second Law. One implication of this result is that as we take steps to reduce/limit antibiotics consumption in populations, even worldwide, resistance will still get worse and worse—however slowly that may be. Nevertheless, a corollary to the First Law points to the path allowing exiting this resistance process. In medicine in particular, the path making it possible to exit drug resistance through the design of drugs capable of withstanding it is given. On the other hand, these features apply equally to resistance occurrence in agriculture. However, further research is needed relative to the application of the characterizations herein presented. Beyond medicine and agriculture, the characterizations presented here govern the natural process of biological resistance in living organisms as a whole and will strengthen our ability to successfully control resistance in various settings, including human medicine, veterinary medicine, agriculture, and beyond. Looking forward, as the First Law governs the rise of resistance while the Second Law governs the growth of it, there is a Third Law (and this was to be expected) which governs the termination of resistance [94].

## Supplementary Materials

The following are available online at https://www.mdpi.com/2076-0817/8/2/73/s1, Table S1: Effectiveness of Medicinal Plants & Proof by Contradiction.
