# Supplementary material for: Characterization of Biological Resistance and Successful Drug Resistance Control in Medicine
Source: Pathogens. 2019 May 31;8(2):73. doi: 10.3390/pathogens8020073 (PMC6631572; doi:10.3390/pathogens8020073)
Supplement: Supplementary file 1 [file pathogens-08-00073-s001.pdf]

Supplementary Material

Effectiveness of Medicinal Plants & Proof by Contradiction

Characterization of Biological Resistance and Successful Drug Resistance Control in Medicine

(Rudolf Fullybright, 2019)

Background

This Supplementary Material is provided as a complement to the main article. It 1) presents supporting evidence that medicinal plants continue to be effective today and 2) introduces mathematical proof by contradiction that medicinal plants have not encountered resistance from pathogens.

1) Select List of 111 Effective Pathogen-Specific Medicinal Plants, Pathogens Targeted, and Literature References Reporting Effectiveness

Table 1 below presents a select list of more than 100 medicinal plants from various geographical regions of the world. Multiple peer-reviewed, published studies have shown those plants to still be effective against their target pathogens today, as of the 21st century, when those studies are published. Table 1 presents only a restricted, partial list of such studies. Far more peer-reviewed, published studies are available in the public domain, literature.

Continuing to be effective in the 21st century and having appeared on earth millions of years ago imply that medicinal plants have remained effective against pathogens throughout their existence on earth, for millions of years.

Table 1: Select list of 111 effective medicinal plants, target pathogens, literature references, and year of effectiveness report.

Plant ID	Plant Name	Region of Origin	Target Pathogens	Published References Reporting Effectiveness	Year Effectiveness is Reported
**1.**	*Terminalia chebula*	India	*Streptococcus mutans, Lactobacillus casei,* or *Staphylococcus aureus*	Gowd Pratap et al. Evaluation of three medicinal plants for anti-microbial activity. Ayu. 2012 Jul-Sep; 33(3): 423–428. doi: 10.4103/0974-8520.108859 PMCID: PMC3665089 PMID: 23723653	2012
**2.**	*Clitoria ternatea*				2012
**3.**	*Wedelia chinensis*				2012
ID	Plant Name	Region	Target Pathogens	References	Report Year
**4.**	*Arum italicum Mill*	Turkey	*Bacillus subtilis, Escherichia coli, Staphylococcus aureus, Pseudomonas aeruginosa, Candida albicans,* or *Aspergillus niger*	Ömer Ertürk. Antibacterial and Antifungal Effects of Alcoholic Extracts of 41 Medicinal Plants growing in Turkey. Czech J. Food Sci. Vol. 28, 2010, No. 1: 53–60	2010
**5.**	*Lathyrus sativus L.*				2010
**6.**	*Cuminum cyminum L*				2010
**7.**	*Aesculus hippocastanum L.*				2010
**8.**	*Jasminium officionale L.*				2010
**9.**	*Thymus capitatus L.*				2010
**10.**	*Viscun album L.*				2010
**11.**	*Ammi visnaga Lam.*				2010
**12.**	*Nigella arvensis L.*				2010
**13.**	*Coriandrum sativum L.*				2010
**14.**	*Ocimum basillicum L.*				2010
**15.**	*Tanecetum sorbifolium*				2010
**16.**	*Achillea biebersteinii*				2010
**17.**	*Buxus sempervirens L.*				2010
**18.**	*Alkanna*				2010

	tinctoria L.				
**19.**	*Pimpinella anisum L.*				2010
**20.**	*Artemisia absinthium L.*				2010
**21.**	*Origanum vulgare L.*				2010
**22.**	*Colutea arborescens L.*				2010
**23.**	*Diospyrus lotus L.*				2010
**24.**	*Erica verticillata Forsk.*				2010
**25.**	*Galega officinalis L.*				2010
**26.**	*Sambucus nigra L.*				2010
**27.**	*Laurus nobilis L.*				2010
**28.**	*Vitex agnus costus L.*				2010
**29.**	*Alhagi camelorum Fisch.*				2010
**30.**	*Pistacia lentiscus L.*		*Aspergillus niger*		2010
**31.**	*Vicia faba L.*		*Bacillus subtilis, Escherichia coli, Staphylococcus aureus, Pseudomonas aeruginosa, Candida albicans,* or *Aspergillus niger*		2010
**32.**	*Liguidamber orientalis*				2010
**33.**	*Rhus coriaria L.*				2010
**34.**	*Prunus laurocerasus L.*				2010
**35.**	*Alnus glutinosa Goertn*				2010
**36.**	*Camelia sinensis L.*				2010
**37.**	*Linum bienne Mill.*				2010
**38.**	*Tamarix*				2010

	smyrensis				
**39.**	*Artemisia santonicum L.*				2010
**40.**	*Scorzonera mollis Bieb.*				2010
**41.**	*Hypericum perforatum L.*				2010
**42.**	*Achillea coarciata Poir.*				2010
**43.**	*Pimenta officinalis Lindl*				2010
**44.**	*Cocos nucifera L.*				2010
ID	Plant Name	Region	Target Pathogens	References	Report Year
**45.**	*Berberis vulgaris*	Algeria	*Staphylococcus aureaus, E. faecalis, E. cloacae*	Mohamed Senouci Bereksi et al. Evaluation of Antibacterial Activity of some Medicinal Plants Extracts Commonly Used in Algerian Traditional Medicine against some Pathogenic Bacteria. Pharmacog J. 2018;10(3):507-12.	2018
**46.**	*Cinnamomum cassia*		*Staphylococcus aureaus, E. faecalis, E. cloacae, E. coli, K. pneumoniae, P. aeruginosa*		2018
**47.**	*Cistus monspeliensis*				2018
**48.**	*Nigella sativa*		*S. aureus*		2018
**49.**	*Punica granatum*		*Staphylococcus aureaus, E. cloacae, E. coli, K. pneumoniae, P. aeruginosa*		2018
**50.**	*Rhus tripartita*		*Staphylococcus aureaus, E. faecalis, E. coli, P. aeruginosa*		2018
**51.**	*Withania frutescens*		*S. aureus*		2018
ID	Plant Name	Region	Target Pathogens	References	Report Year
**52.**	*Azadirachta indica*	India	*Herpes Simplex Virus-1 (HSV-1), Coxsackievirus virus B-4,* Aspergillus *sp.,* Rhizopus *sp., Curvularia lunata, H. pennisetti, C.*	Mohammad A. Alzohairy. Therapeutics Role of Azadirachta indica (Neem) and Their Active Constituents in Diseases Prevention and Treatment. Evid	2016

			gloeosporioides f. sp. Mangiferae, Alternaria solani, Cladosporium, Plasmodium berghei, Streptococcus salivarius Fusobacterium nucleatum	Based Complement Alternat Med. 2016; 2016: 7382506. Published online 2016 Mar 1. doi: 10.1155/2016/7382506	
			Escherichia coli O157:H7	Subbarao V. Ravva and Anna Korn. Effect of Neem (Azadirachta indica) on the Survival of Escherichia coli O157:H7 in Dairy Manure. Int J Environ Res Public Health. 2015 Jul; 12(7): 7794–7803. Published online 2015 Jul 10. doi: 10.3390/ijerph120707794.	2015
			Smallpox, Chicken pox, Poxvirus, Herpes viruses, Poliomyelitis	Zeinab Nazarian Samani and Mahmoud RaRfieian Kopaei. Effective medicinal plants in treating Hepatitis B. Int J Pharm Sci & Res 2018; 9(9): 3589-96. doi: 10.13040/ IJPS.0975-8232.9(9).3589-96	2018

ID	Plant Name	Region	Target Pathogens	References	Report Year
**53.**	*Acacia catechu*	Thailand	*enterohaemorrhagic Escherichia coli (EHEC) O157:H7*	Voravuthikunchai S. et al. Effective medicinal plants against enterohaemorrhagic Escherichia coli O157:H7. J Ethnopharmacol. 2004 Sep;94(1):49-54. PMID: 15261962 DOI: 10.1016/j.jep.2004.03.036	2004
**54.**	*Holarrhena antidysenterica*				2004
**55.**	*Peltophorum pterocarpum*				2004
**56.**	*Psidium guajava*				2004
**57.**	*Quercus infectoria*				2004

**58.**	*Uncaria gambir*				2004
**59.**	*Walsura robusta*				2004
ID	Plant Name	Region	Target Pathogens	References	Report Year
**60.**	*Solanum nigrum*	Iran	*S. aureus, Listeria monocytogenes,* and *Vibrio cholera*	Rahnama M, Fakheri B A, Mashhady M A, Saeidi S, Jahani S. The Antimicrobial Effects of Medicinal Plants on Pathogenic Food Bacteria, Int J Infect. 2017 ; 4(2):e40238. doi: 10.5812/iji.40238	2016
**61.**	*Mentha longifolia*		*Bacillus cereus*		2016
**62.**	*Mentha piperita*		*Bacillus cereus* and *Vibrio cholera*		2016
**63.**	*Withania somnifera*		*Bacillus cereus* and *Shigella dysenteriae*		2016
ID	Plant Name	Region	Target Pathogens	References	Report Year
**64.**	*Grapes*	Canada	Herpes simplex virus (HSV), Poliovirus type 1, Coxsackie virus B5, and Echovirus	Konowalchuk J and Speirs JI: Virus inactivation by grapes and wines. Applied and Environmental Microbiology 1976; 32: 757-763	1976
**65.**	*Apple*			Konowalchuk J and Speirs JI: Antiviral activity of fruit extracts. Journal of Food Science 1976b; 41: 1013-1017	1976
**69.**	*Strawberry*			Konowalchuk J and Speirs JI: Antiviral effect of apple beverages. Applied and Environmental Microbiology 1978a; 36: 798-801	1978
				Konowalchuk J and Speirs JI: Antiviral effect of commercial juices and beverages. Applied and Environmental Microbiology 1978b; 35: 1219-1220	1978
ID	Plant Name	Region	Target Pathogens	References	Report Year
**70.**	*Melaleuca*	*Australi	HSV-1	Zeinab Nazarian	2018

	alternifolia	a		Samani and Mahmoud RaRfieian Kopaei. Effective medicinal plants in treating Hepatitis B. Int J Pharm Sci & Res 2018; 9(9): 3589-96. doi: 10.13040/ IJPS.0975-8232.9(9).3589-96	
**71.**	*Santolina insularis*	Europe	HSV-1, HSV-2, herpes types		2018
**72.**	*Santalum album*	Sri Lanka	HSV-*1*		2018
**73.**	*Cardamine angulata*	California, USA			2018
**74.**	*Polypodium glycyrrhiza*	North America			2018
**75.**	*Verbascum Thapsus*	Europe, Africa			2018
**76.**	*Conocephalum conicum*	Mediterranean region			2018
**77.**	*Lysichiton americanum*	N. America			2018
**78.**	*Sanicula europaea*	Europe	Influenza		2018
**79.**	*Nigella sativa*	SW Asia	murine cytomegalovirus (MCMV)		2018
**80.**	*Eleutherococcus senticosus*	Siberia	DNA viruses, respiratory syncytial virus (RSV) adenovirus		2018
**81.**	*Rosa nutkana*	Pacific NW	Enteric corona virus		2018
**82.**	*Amelanchier alnifolia*	N. America	Enteric corona virus		2018
**83.**	*Ipomopsis aggregate*	N. America	Parainfluenza virus type 3		2018
**84.**	*Lomatium dissectum*	N. America	*Rotavirus*		2018
**85.**	*Potentilla arguta*	N. America	Respiratory syncytial virus (RSV)		2018
**86.**	*Sambucus racemosa*	N. America	Respiratory syncytial virus (RSV)		2018
**87.**	*Dianella longifolia* var . grandis	Australia	Poliovirus type 1		2018
**88.**	*Pterocaulon sphacelatum*	N. & S. America, Australia	Poliovirus type 1		2018
**89.**	*Euphorbia*	Australia	Human		2018

	australis		cytomegalovirus (HCMV)		
**90.**	*Scaevola spinescens*	Australia	Human cytomegalovirus (HCMV)		2018
**91.**	*Eremophila latrobei* subsp. Glabra	Australia	Ross River virus (RRV)		2018
**92.**	*Pittosporum phylliraeoides* var. Microcarpa	Australia	Ross River virus (RRV)		2018
**93.**	*Sanicula europaea*	Europe	Parainfluenza virus type 2		2018
**94.**	*Myrcianthes cisplatensis*	Central & S. America	RSV, Adenovirus serotype7‘ HSV-1		2018
**95.**	*Opuntia streptacantha*	Americas	HSV, equine herpes virus, pseudorabies virus, influenza virus		2018
**96.**	*Bergenia ligulata*	Himalayas	Influenza virus, HSV		2018
**97.**	*Nerium indicum*	India			2018
**98.**	*Holoptelia integrifolia*	India			2018
**99.**	*Curcuma longa* Linn.	SE Asia	Hepatitis B Virus		2018
**100.**	*Ganoderma lucidum*	Worldwide			2018
**101.**	*Phyllanthus amarus*	C. America			2018
**102.**	*Acanthus ilicifolius* L	Australia			2018
**103.**	*Acacia nilotica*	Africa, Middle East, India	Hepatitis C Virus		2018
**104.**	*Zingiber officinale*	SE Asia			2018
**105.**	*Silybum marianum*	Worldwide			2018
**106.**	*Quercus infectoria*	S. Europe			2018

ID	Plant Name	Region	Target Pathogens	References	Report Year
		& Middle East			
**107.**	*Ocimum basilicum*	Tropical Africa / Asia	Hepatitis A Virus		2018
**108.**	*Taraxacum Officinalis*	Europe & Asia	Hepatitis		2018
**109.**	*Morinda citrifolia*	SE Asia, Australia			2018
**110.**	*Matricaria chamomilla*	Europe			2018
**111.**	*Panax ginseng*	Korea, Japan	*Helicobacter pylori, Pseudomonas aeruginosa, Staphylococcus aureus, Porphyromonas gingivalis, Listeria monocytogenes, Bacillus cereus, Streptococcus pneumoniae*	Ye-Ram Kim and Chul-Su Yang. Protective roles of ginseng against bacterial infection. Microb Cell. 2018 Nov 5; 5(11): 472–481. doi: 10.15698/mic2018.11.654 & ***references therein.***	2018
ID	Plant Name	Region	Target Pathogens	References	Report Year

**: for plant species ID 70 through to 111, Wikipedia was used as the source establishing region of nativity.*

Table 1 above presents a limited sample of over 100 medicinal plants from around the world which studies have found to still be effective against their target pathogens as of the 21st century. Let us now examine mathematical proof by contradiction that medicinal plants have encountered no resistance from pathogens.

2) Proof by Contradiction of Non-existence of Resistance to Medicinal Plants

In Mathematical Logic, there is a concept termed *Proof by Contradiction*: proving that something exists by proving that its opposite does not exist—and vice versa.

We are giving here proof, using the mathematical logic concept of proof by contradiction,

that, in addition to the non-observation of resistance to any medicinal plant, medicinal plants have encountered no resistance from pathogens in their millions of years of existence.

Courtesy of the Computational Geometry Laboratory of the School of Computer Science at McGill University, Montréal, Québec, Canada, we have a very good description of what is Proof by Contradiction [1]. For the purposes of this analysis, we are going to have to take a look at a number of extra sources, such as Chapter 62 (*Surveillance of Antiretroviral Drug Resistance in Resource-poor Settings* by Inge Derdelinckx and Charles Boucher) of *Global HIV/AIDS Medicine*, 2008, Pages 703-710. That reference infers how "effectiveness" and "resistance" are **opposites** of each other: As one goes up, the other goes down; if one has one, then one does not have the other.

In fact, here is what the Conclusion of Chapter 62 states:
"*Antiretroviral drug resistance is an inevitable consequence when providing long-term treatment and should not be seen as a limitation for providing antiretrovirals to patients in resource-poor settings. The rapid expansion of HIV/AIDS treatment access is an urgent public health necessity. However, efforts should be undertaken to reduce the development of drug resistance as much as possible by providing healthcare infrastructures that will* ***maximize*** *the* ***effectiveness*** *of treatment and* ***minimize*** *the risk for* ***drug resistance****. …***"** (emphasis added).

We also need to take a look at what the US Centers for Disease Control and Prevention (US CDC) has to say about "effectiveness" and "resistance," stating: "*If antibiotics* ***lose*** *their* ***effectiveness****, then we* ***lose*** *the ability to treat infections and* ***control*** *public health threats.*" [2] That statement implies that if effectiveness is lost, then resistance has appeared. Conversely, it also means that if there is effectiveness, then there is no resistance.

And here is a statement from the World Health Organization (WHO) regarding **"**effectiveness**"** and **"**resistance**": "***To* ***maximize*** *the long-term* ***effectiveness*** *of first-line ART regimens, and to ensure that people are taking the most effective regimen, it is essential to continue*

monitoring ***<u>resistance</u>*** *and to* ***<u>minimize</u>*** *its further emergence and spread.*" [3].

That statement, like the previous ones from the other sources, equally implies and means that "effectiveness" and "resistance" **are opposites of each other**, are **the opposite sides of the same coin**, and that **if you have one, then you do not have the other**. In fact, "effectiveness" means that the drug is effective, that **it kills the pathogen.** And "resistance" means that the pathogen resists the drug and **is NOT killed by it.** So, "effectiveness" and "resistance" are opposites of each other and **cancel each other out.** Both **do <u>not</u>** and **<u>cannot</u>** exist simultaneously. **It is <u>impossible</u> to have both at the same time.** So, proving the existence of one is equal to proving the non-existence of the other.

One may be tempted to think that lack of observation of resistance to a medicinal plant is not evidence per se. However, we need to consider the ten (10) published references mentioned in the main article (References [27] to [36]), in addition to the published references of Table 1 above, reporting more than 100 medicinal plants as being still effective against their target pathogens, together with the dozens more published references not listed in Table 1 (which cannot be listed because of space restrictions), and which all prove that medicinal plants continue to be effective against their target pathogens today. Again, beyond the 10 publications presented in the main article, and in addition to the more than 100 medicinal plants listed in Table 1 above, along with their own supporting references, dozens of other publications are out there, in the public domain, all reporting continued effectiveness of hundreds of medicinal plants from around the world, as of this 21st century, and regardless of geographic origin.

With that foundation laid down, let us now move on to the mathematical concept of proof by contradiction.

The **<u>McGill University Proof by Contradiction reference</u>** given above states:
"*We now introduce a third method of proof, called proof by contradiction. This new method is not limited to proving just conditional statements – it can be used to prove any kind of statement*

whatsoever. The basic idea is to assume that the statement we want to prove is false, and then show that this assumption leads to nonsense. We are then led to conclude that we were wrong to assume the statement was false, so the statement must be true."

On the basis of all of the above, it is established that the statement we want to prove is:
Statement A: **"**Medicinal plants **have encountered no resistance** from pathogens.**"**
The opposite of Statement A is Statement B.
Statement B: **"**Medicinal plants **have encountered resistance** from pathogens**"** which **is equal to: "**Medicinal plants **have lost their effectiveness** against pathogens.**"**

The Proof by Contradiction reference cited above says to assume that the statement we want to prove is false. So we assume that Statement A is false.

However, we have seen from the multiple sources above (including the US Centers for Disease Control and the World Health Organization) that "effectiveness" and "resistance" **are opposites of each other** and that **having one means not having the other**.
Therefore, if Statement A is false, then Statement B is true and that means that medicinal plants **have encountered resistance** from pathogens, **which also means that medicinal plants have lost their effectiveness** against pathogens.

But we have the ten studies presented in References [27] through [36] in the main article along with hundreds more studies in the public domain, published by additional researchers, freely available on the Internet, and which state and confirm that **medicinal plants are still effective against their respective target pathogens today.**

However, Statement B says that medicinal plants have lost their effectiveness, which is in contradiction with the findings of those studies. Therefore, the **assumption** that Statement A is false **is wrong** and, therefore, Statement A **is true**.
Therefore, medicinal plants **have encountered no resistance** from pathogens.

Conclusion: Medicinal plants **have encountered no resistance** from pathogens.

As seen earlier, "effectiveness" and "resistance" are opposites of each other and having one necessarily implies not having the other. Consequently, by proving that medicinal plants are presently effective against the pathogens, those studies have thereby proven that there is no resistance to the plants. In Mathematical Logic, the proof of effectiveness given by those studies is equally proof of non-existence of resistance to the plants.

References:

63. McGill University. Proof by Contradiction. Computational Geometry Laboratory. School of Computer Science. McGill University. Montreal, Canada. 2019. Retrieved May 2019 from: http://cgm.cs.mcgill.ca/~godfried/teaching/dm-reading-assignments/Contradiction-Proofs.pdf
64. US CDC. Antibiotic / Antimicrobial Resistance (AR / AMR) – About Antimicrobial Resistance. US Centers for Disease Control and Prevention. 2019. Retrieved May 2019 from: https://www.cdc.gov/drugresistance/about.html
65. World Health Organization. Global trends – other microbes. WHO, 2019. Copenhagen, Denmark. Retrieved May 2019 from: http://www.euro.who.int/en/health-topics/disease-prevention/antimicrobial-resistance/about-amr/global-trends-other-microbes
